# The impact of the COVID‐19 pandemic on the diagnosis and treatment of skin cancers, a mini review

**DOI:** 10.1002/cnr2.1854

**Published:** 2023-07-12

**Authors:** Zeinab Aryanian, Kamran Balighi, Azadeh Goodarzi, Ali Sadeghi Nia, Zeinab Mohseni Afshar, Parvaneh Hatami

**Affiliations:** ^1^ Autoimmune Bullous Diseases Research Center Tehran University of Medical Sciences Tehran Iran; ^2^ Department of Dermatology Babol University of Medical Sciences Babol Iran; ^3^ Department of Dermatology, Razi Hospital Tehran University of Medical Sciences Tehran Iran; ^4^ Department of Dermatology, Rasool Akram Medical Complex Clinical Research Development Center, School of Medicine Iran University of Medical Sciences Tehran Iran; ^5^ Skin and Stem Cell Research Center Tehran University of Medical Sciences Tehran Iran; ^6^ Clinical Research Development Center, Imam Reza Hospital Kermanshah University of Medical Sciences Kermanshah Iran; ^7^ Behayand Cancer Team Tehran Iran

**Keywords:** BCC, COVID‐19, melanoma, premalignant lesions, SCC, skin cancer

## Abstract

**Background:**

The SARS‐Cov‐2 pandemic has caused dermatologists around the world to adapt their practice in the aim of protecting patients with special clinical conditions such as those having skin cancers or premalignant conditions and some diagnostic or therapeutic programs would be suspended due to being infected to SARS‐CoV‐2. We focused the existing data in this regard through searching on PubMed, Google Scholar, and Scopus and tried to summarize recommendations for treating this group of patients in COVID era to provide a practical guide for clinicians.

**Recent findings:**

The number of diagnosed skin cancers dropped significantly since the beginning of the pandemic, specially in the peaks of various variants of the SARS‐CoV‐2 infection. The new guidelines allowed even a 3‐month delay in excision and recommended surgery for non‐melanoma growing skin cancers.

**Conclusion:**

Dermatologists should perform a careful, individualized risk–benefit assessment for their patients and consider some changes in routine protocols such as having a delayed diagnostic or therapeutic intervention.

## 1 INTRODUCTION

At the beginning of the coronavirus disease 2019 (COVID‐19) pandemic, many non‐urgent visits to outpatient clinics had been deferred. This had led to significant pitfalls in the diagnosis and management of infectious and noninfectious diseases.^1^ Skin cancers are among the conditions which have been impacted significantly in the current pandemic due to their delayed diagnosis, which has led to increased healthcare costs, morbidity and mortality.^2^ Here, we present a brief review of skin cancers challenges in the COVID era.

### 1.1 Diagnostic challenges

As has been reported in different studies, the number of diagnosed cases of skin cancers, including cutaneous squamous cell carcinoma (SCC) and basal cell carcinoma (BCC) dropped significantly since the beginning of the pandemic, compared to the corresponding time before the pandemic.^3^ This decrease in the diagnosis became more prominent in the peaks of various variants of the SARS‐CoV‐2 infection. The decrease in the incidence of skin cancers since the beginning of the pandemic can be attributed to the reduction in the diagnosis of cases, rather than the decreased incidence.^4^ However, some authorities reported higher rates of skin cancer detection during the COVID era, which implies the continuous skin cancer services even during the strict shutdown.^5^

The patients' reluctance to visit practitioners and dermatologists has been the main reason for diagnostic delay of skin cancers in recent years. However, the health system also contributes in this delay; social distancing might have led to decreased access to health care systems. Skin biopsy rates had drastically reduced due to the pandemic. This decrease had been more prominent among females, older age and in the beginning of the lockdown. Moreover, this decrease had been greater for keratinocyte carcinoma than melanoma.^6^

The delayed diagnosis has led to significant consequences; the Breslow thickness of skin cancers has been increased and local invasion has been more common at the time of diagnosis.^7^ Therefore, stage is more progressed with higher severity and more probability of metastasis at the time of diagnosis, thereby affecting the survival.^8^, ^9^ This has also resulted in more skin biopsies performed by plastic surgeons, compared to dermatologist due to the presence of advanced lesions.^6^ All these has led to diagnostic challenges since suspicion per se cannot detect the type of a cancer; a lesion thought to be BCC could be melanoma, each with quite different prognosis. Moreover, dermoscopic examination which is a major aid in making an early diagnosis of skin cancers is also restricted due to the shutdown and social distancing.^10^

Telemedicine is another issue that has been highlighted during the COVID era. This strategy can limit unnecessary hospital visits and help in the follow‐up of dermatologic disorders. Teledermatology can also be utilized to screen those individuals suspected to skin cancers.^11^ However, this tool can be partially beneficial in detecting skin cancers as it can be used in first‐level screening of melanoma and non‐melanoma skin cancers (NMSC) but assessment of some pigmented lesions needs face‐to‐face visits and the definite diagnosis needs biopsy.^12^, ^13^

On the other hand, SARS‐CoV‐2 infection in a patient with preexisting cancer, either diagnosed and being managed or undiagnosed, is supposed to lead to greater morbidity and mortality and affect the infection outcome like any other comorbidity.^14^ Moreover, targeted treatments such as immunotherapies can impact the course of SARS‐CoV‐2 infection.^15^ However, the issue has been quite different for skin cancers, particularly BCC. The reason might be the higher vitamin D levels in these cancers which prevents infection or improves the outcome in COVID patients.^16^

## 2 DISCUSSION

The COVID‐19 pandemic not only has had a significant impact in the diagnosis of skin cancers, but also has affected its management considerably. The recommendations to stay at home and also the patients' preference to minimize clinic visits are some reasons for delayed therapeutic measures.^17^

In general, antineoplastic treatments should be carried out in a timely and continuous manner even in the COVID era unless for cases with specific tumor and clinical characteristics for which the treatment can be postponed.^18^ In fact, skin malignant lesions should be categorized into the ones needing urgent therapeutic intervention and those not being very emergent. The histopathologic examination, and tumor location and size are considered in the decision making for urgent management. Some skin cancers and pre‐malignant conditions can be managed non‐surgically and only with the aim of topical treatment in the COVID era with strict lockdown. Actinic keratosis (AK), Bowen disease (BD), and superficial basal cell carcinomas (BCCs) are examples of lower risk conditions. However, some skin cancer types such as melanoma require more rigid interventions.^19^

Photodynamic therapy (PDT) is an important therapeutic option for some types of low risk skin cancers. However, this modality and some other topical treatments have been suspended in the shutdown period, affecting the management of skin cancers. In turn, other alternative topical treatments such as 5‐fluorouracil and imiquimod have been applied.^20^ However, a certain type of treatment was more commonly administered in each wave of SARS‐CoV‐2 infection. For example, systemic oral agents such as BRAF/MEK inhibitors had decreased the frequency of clinic visits during the pandemic.

Considering radiation as a main component of therapeutic regimen of skin cancers, it has been suggested to use modified fractionation radiotherapy for those skin cancers needing definitive radiation, particularly melanoma. However, it is possible to postpone radiotherapy for 2 to 3 months in the treatment of lentigo maligna, lentigo maligna melanoma, and melanoma in situ.^21^

On the other hand, advanced skin cancers care including surgery and wide local excisions, has been a great challenge during the COVID‐19 pandemic.^22^ Moreover, follow‐up visits have been suspended due to the lockdown, which further impacted the cancer surveillance. Since early surgical excision is the treatment of choice for malignant melanoma, the postponement of elective surgeries in the COVID era had led to therapeutic pitfalls for this type of skin cancer, particularly the metastatic ones.^23^ The new guidelines allowed even a 3‐month delay in excision and recommended surgery only for non‐melanoma growing skin cancers.^24^ This is in contrast with the National Comprehensive Cancer Network (NCCN) guidelines which acknowledge that skin cancers outcome is greatly affected by any delay in tumor surgical removal and recommend melanomas to be immediately excised at the diagnosis. Moreover, high risk BCCs (locally aggressive, larger than 0.5 cm, or those located on high‐risk areas of the face like palpebral and lip), and rapidly growing SCCs also require urgent oncologic surgery.^25^ Therefore, plastic surgeons should make decisions to prioritize surgery for more aggressive forms of skin cancers, including melanomas with the need to be re‐excised or undergo sentinel node biopsy (SNB), ulcerated skin cancers with the risk of hemorrhage, and skin metastases of remote solid tumors.^26^ In general, it is advised not to delay surgical diagnostic or therapeutic interventions for more than 3 months in melanoma skin cancers.^27^

Although skin surgical procedure is believed to be a low‐to‐medium risk surgery, the surgeon might be at risk of acquiring SARS‐CoV‐2 infection from an infected patient especially during the interventions on the face in which the patient cannot wear a mask.^28^ Therefore, many centers announced the rule for being screened for SARS‐CoV‐2 infection by means of RT‐PCR test in skin cancer patients who are planned to undergo surgery. The surgery is delayed for those patients who have positive tests.^29^ Some guidelines even acknowledge the need to be swab PCR test negative for SARS‐CoV‐2 in any radiotherapeutic, chemotherapeutic, immunotherapeutic and surgical intervention.^30^

Follow‐up visits are essential components of cancer management. Skin cancers should also be regularly monitored following surgery or medical therapy. In the time of lockdown, such follow‐up visits might be suspended, held with longer intervals or handled by teledermatology. However, it should be emphasized that any atypical melanocytic lesion, fast growth or rapid color or shape change should be considered significant and needs dermatologist's visit as soon as possible.^31^

Another important point is the decision to cease or continue cancer treatment in patients who develop symptoms of COVID‐19. These patients are recommended to undergo a definitive diagnostic test before suspending their oncologic treatment since some immunotherapeutic agents can induce COVID‐19 like manifestations which are difficult to differentiate from COVID‐19.^10^ However, Hedgehog (HHI) pathways inhibitors like vismodegib and sonidegib have side effects with less interfering symptoms.^32^ In case of definitive SARS‐CoV‐2 infection, it is reasonable to suspend some targeted therapies like BRAF/MEK inhibitors and HHIs as they aggravate the course of SARS‐CoV‐2 infection and affect the outcome.^15^ Anti‐PD‐1 courses are usually postponed in the settings of an acute infection due to the probability of COVID‐19 induced cytokine storm.^33^ It is advised to delay cancer treatment for a short 2 to 6 weeks period or until clinical improvement.^34^ However, metastatic melanoma patients who are on immunotherapy or target therapy have better continue their cancer treatment despite being infected with SARS‐CoV‐2 unless they have developed respiratory dysfunction.^35^

## 3 CONCLUSION

The COVID pandemic has imposed a great burden on all aspects of human health, including cancers. The number of diagnosed cases of skin cancers dropped significantly since the beginning of the pandemic, especially in the peaks of various variants of the SARS‐CoV‐2 infection. The patients' reluctance to visit practitioners and dermatologists has been the main reason for diagnostic delay of skin cancers in recent years which has been more prominent among females and older age. Skin malignant lesions should be categorized into the ones needing urgent therapeutic intervention and those not being very emergent by considering he histopathologic examination, tumor location and size. The new guidelines allowed even a 3‐month delay in excision and recommended surgery only for non‐melanoma growing skin cancers. In the time of lockdown, such follow‐up visits might be suspended, held with longer intervals or handled by teledermatology. In case of definitive SARS‐CoV‐2 infection, it is reasonable to suspend some targeted therapies for a short 2–6 weeks period or until clinical improvement. The exceptions are metastatic melanoma patients who are on immunotherapy or target therapy unless they have developed respiratory dysfunction.

Overall, any decision to delay diagnostic or therapeutic intervention for a suspected or confirmed skin cancer should be made after consulting and discussing with dermatologists and weighing the risk‐benefits.

## AUTHOR CONTRIBUTIONS

Zeinab Aryanian, Kamran Balighi, Azadeh Goodarzi, Ali Sadeghi Nia, Zeinab Mohseni Afshar, Parvaneh Hatami contributed equally to the preparation of data and finalization of this article.

## ETHICS STATEMENT

The protocol of this study was approved by the ethics committee of Tehran University of Medical Sciences.

## FUNDING INFORMATION

The authors received no funding for this project.

## CONFLICT OF INTEREST STATEMENT

All the authors declare that there is no conflict of interest.

## Data Availability

The data that support the findings of this study are available from the corresponding author upon reasonable request.
